# Association between roentgenographic findings of the cervical spine and neck symptoms in a Japanese community population

**DOI:** 10.1007/s00776-014-0549-8

**Published:** 2014-02-26

**Authors:** Gentaro Kumagai, Atsushi Ono, Takuya Numasawa, Kanichiro Wada, Ryo Inoue, Hiroki Iwasaki, Kaori Iwane, Masashi Matsuzaka, Ippei Takahashi, Takashi Umeda, Shigeyuki Nakaji, Yasuyuki Ishibashi

**Affiliations:** 1 Department of Orthopaedic Surgery, Hirosaki University Graduate School of Medicine, 5 Zaifu-cho, Hirosaki, Aomori 036-8562 Japan; 2 Department of Social Medicine, Hirosaki University Graduate School of Medicine, 5 Zaifu-cho, Hirosaki, Aomori 036-8562 Japan

## Abstract

**Background:**

Radiographic findings may provide clues to the underlying cause of neck symptoms. However, these associations remain controversial. This study investigates the association between roentgenographic findings of the cervical spine and neck symptoms in a Japanese community population.

**Methods:**

A total of 762 volunteers participated in this study. Sagittal radiographs of the cervical spine were taken and a questionnaire about the presence of and visual analog scale (VAS) for neck pain or stiff shoulder was completed. The sagittal alignment of the cervical spine (C2–C7) and the degenerative index were measured from lateral aspect radiographs. Three groups based on the sagittal alignment of C2–C7 were defined: straight-spine, lordotic-spine, and kyphotic-spine. The roentgenographic findings were examined in relation to symptoms.

**Results:**

The prevalence rate of stiff shoulder on the day of examination was significantly higher in females than males. Although the VAS for neck pain and stiff shoulder on the examination day and for stiff shoulder in the preceding 12 months were not significantly different between females and males, that for neck pain in the preceding 12 months was significantly higher in females than males. Although there was no association between the sagittal alignment of C2–C7 and neck symptoms in males or females, a significant correlation between the degenerative index and VAS for neck pain on the examination day and in the preceding 12 months was seen in females after adjusting for age. The prevalence of and VAS for neck pain and stiff shoulder were not significantly different among the three C2–C7 sagittal alignment groups.

**Conclusion:**

Although the sagittal alignment of the cervical spine was not associated with neck symptoms, degenerative changes were associated with the severity of neck pain in females.

## Introduction

Low-back and neck pain are critical public health problems. The prevalence of neck pain in the general population is 10–15 % [1–3], and the 3-month prevalence of back and/or neck pain in the US is reported to be 31 % (low-back pain only: 34 million, neck pain only: 9 million, both back and neck pain: 19 million) [4]. The total cost of neck pain in the Netherlands in 1996 was estimated to be US $686 million [5].

Radiographic findings may provide clues to the underlying cause of neck symptoms. The finding that degenerative changes of the cervical spine are common in asymptomatic individuals challenges the notion of cause and effect [6]. With age, the number of subluxations and the incidence and severity of degenerative changes increase [7, 8]. The most typical changes include osteoarthritis of the facets with reduced joint space and disc narrowing. Some studies have identified a relationship between roentgenographic findings of the cervical spine and neck symptoms [8, 9]. On the other hand, some reports have shown that there is no significant relationship between degenerative changes and pain [7, 10, 11].

Degenerative changes of the cervical spine are often accompanied by a shortening of the anterior or posterior vertebral column [12, 13], which alters the sagittal profile of the cervical spine [7]. The relationship between sagittal alignment and neck symptoms also remains controversial [14, 15]. While these studies examined symptomatic patients or volunteers, there have been few general community population-based studies. Therefore, we conducted a general-population-based, cross-sectional research study of the cervical spine in 762 participants, in 2008. The objective of the present study was to investigate the association between roentgenographic findings of the cervical spine and neck symptoms in a Japanese community population.

## Materials and methods

### Participants

The Iwaki Health Promotion Project is a community-based program with the goal of increasing average life expectancy by performing general health checkups. This program began in 2005 and is being conducted over a 10-year period. About 1,000 people aged 20 years or more and living in the Iwaki area of Hirosaki city, located west of Aomori, Japan participate every year. Physicians, surgeons, orthopedists, gynecologists, urologists, psychiatrists, dermatologists, and dentists from Hirosaki University are involved in investigating various diseases and disorders [16]. Our research on the association between roentgenographic findings of the cervical spine and neck complaints is one part of this project.

A total of 886 people (20–86 years old, 325 males and 561 females) participated in the Iwaki Health Promotion Project in 2008. Exclusion criteria for our study were a history of previous trauma of the cervical spine or systemic disease involving the cervical spine (e.g., rheumatoid arthritis). Patients with neck complaints under treatment and those taking oral nonsteroidal anti-inflammatory drugs (NSAIDs) were also excluded. Eventually, 762 participants (283 males and 479 females) were included in our study. The mean (±SD) ages of the male and female participants were 57.8 ± 12.5 and 58.7 ± 10.9 years, respectively, with no difference between males and females (*P* = 0.376) (Table 2). The participants were informed verbally and in writing about the purpose of the study and the methods used for data collection. We also informed them that they could discontinue their participation and their anonymity would be protected, and obtained their written consent. The Iwaki Health Promotion Project is being conducted with the approval of the ethics committee of the Hirosaki University Graduate School of Medicine.

All participants filled out questionnaires about their medical history, lifestyle, smoking, drinking, fitness habits, occupational history, family history, and health-related quality of life, and provided specific information about various diseases, including neck symptoms. Anthropometric measurements included height, weight, body mass index (BMI), body fat percentage, bone density, waist-hip ratio, bilateral grip strength, functional reach test, and timed up and go test. Blood and urine samples were taken from all the participants before breakfast in the early morning for biochemical examinations. In a clinical examination by a highly experienced orthopedist, information about the knees, hips, elbows, neck, and lower back, including range of motion, was obtained. Plain radiographs of the knees, hips, hands, cervical spine, and lumbar spine were taken for 817 of the 886 participants.

### Neck symptoms

All the participants filled out questionnaires about whether they experienced neck pain or stiff shoulder on the day of examination or even once in the previous 12 months. Stiff shoulder, which is common in Japan, was defined as “myotonia, heaviness, and dull pain in the cervical to scapular regions’’ [17]. To evaluate the degree of neck pain and stiff shoulder, a visual analog scale (VAS) was used. To evaluate the VAS results, the degree of neck pain and stiff shoulder on the examination day or in the preceding 12 months was quantified from 0 to 100 mm.

### Radiological assessment

**Fig. 1:**
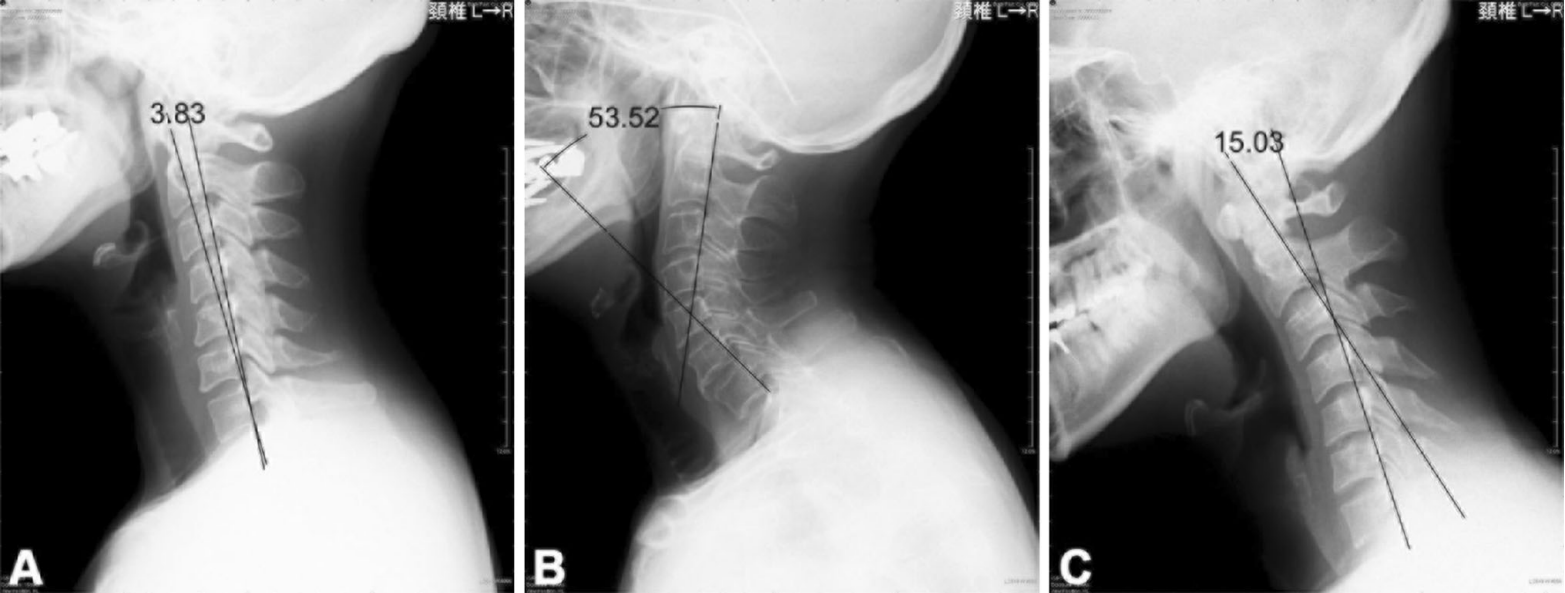
Tangent lines at the posterior vertebral body margins (here of C2 and C7). **a** Straight (−4 to +4°): 48-year-old woman, C2–C7 3.83°. **b** Lordotic (less than −4°): 75-year-old man, C2–C7 53.52°. **c** Kyphotic (more than +4°): 27-year-old man, C2–C7 −15.03°

On the day of the general health checkup, lateral aspect radiographs of the cervical spine were taken with each participant standing in a comfortable position, looking straight ahead, resting the shoulder against the film cassette [7, 8]. Sagittal alignment of the cervical spine (C2–C7) was measured from the radiographs, using the posterior tangent method of the odontoid process and the C7 vertebral body, and three groups were defined: straight-spine (−4° to +4°), lordotic-spine (less than −4°), and kyphotic-spine (more than +4°) [15] (Fig. 1). All the radiographs were measured using CANVAS 8 accurate to 0.1° (Deneba System, Inc., Arlington, Florida, USA).

The degenerative index was determined by Gore’s method [7]. The four principal roentgenographic components of intervertebral disc degeneration—disc space narrowing, endplate sclerosis, and osteophyte formation both anterior and posterior—were recorded for each disc space. A numerical grading system was designed and used to assess the severity of degenerative changes at each of five intervertebral spaces (C2/3–C6/7 level) (Table 1). In 629 participants, C7/T1 could not be seen clearly, so the degenerative changes at that level were not determined. The degenerative index ranged from 0 to 72. The C2–C7 sagittal alignment and degenerative index were measured by two observers, and the interobserver reliability was calculated. The second observer was blinded to the findings of the first observer. Reliability was determined in terms of the interclass correlation coefficient [18].

**Table 1:** Criteria for grading the degenerative changes in the cervical spine at each of five intervertebral spaces (degenerative index)

Numerical value	Disc space narrowing	Vertebral body end-plate sclerosis	Osteophyte formation both anterior and posterior
0	None	None	None
1	25 % decrease	Barely visible	Barely visible
2	50 % decrease	Moderate	Moderate size
3	75 % decrease	Severe	Large

The degenerative index has a possible range of 0–72

% decrease means the ratio to normal disc space narrowing

### Statistical analysis

Data input and calculations were performed using SPSS ver.12.0J (SPSS Inc., Chicago, IL, USA). Comparisons of the characteristics, the sagittal alignment of C2–C7, and the degenerative index between males and females was performed using the Mann–Whitney *U* test. The prevalence rate of neck pain or stiff shoulder was analyzed using a *χ* ^2^ test. The relationships between the sagittal alignment of C2–C7 or the degenerative index and age, or the VAS for neck pain or stiff shoulder in males and females were analyzed by Spearman’s rank correlation coefficient, and partial correlation analysis was performed to adjust the data for age. The VAS results for neck pain and stiff shoulder among the straight, lordotic, and kyphotic groups were compared using analysis of covariance (ANCOVA) and the Bonferroni method after adjusting for age in females. In all analyses, *P* values <0.05 were considered significant.

## Results

### Gender differences in roentgenographic findings and neck symptoms (Table 2)

The mean BMI was 23.4 ± 2.7 (kg/m^2^) in males and 22.7 ± 3.3 (kg/m^2^) in females, which was significantly different (*P* < 0.001). The mean degree of the sagittal alignment of C2–C7 was 18.1 ± 13.3 (range −30.5–63.2). The intraclass correlation coefficient for measurements of the sagittal alignment of C2–C7 was 0.933, and high interobserver reliability was observed. The mean sagittal alignment of C2–C7 was 18.6 ± 13.2 in males and 17.8 ± 13.3 in females, which was not significantly different (*P* = 0.163). The mean degenerative index was 13.8 ± 7.1 (range 0–45). The intraclass correlation coefficient for measurements of the degenerative index was 0.929, and high interobserver reliability was observed. The mean degenerative index was 14.7 ± 8.0 in males and 13.3 ± 6.4 in females, which was a significant difference (*P* = 0.033).

**Table 2:** Age, age groups (20s–30s, 40s–50s, and 50s–80s), BMI, degree of sagittal alignment of C2–C7, and degenerative index of radiographic findings of the cervical spine, and neck symptoms in study participants

	Male (*n* = 283)	Female (*n* = 479)	*P* values
Age (years)	57.8 ± 12.5	58.7 ± 10.9	0.376
20s–30s	20	17	–
40s–50s	132	235	–
60s–80s	131	227	–
BMI (kg/m^2^)	23.4 ± 2.7	22.7 ± 3.3	<0.001*
Sagittal alignment of C2–C7 (°)	18.6 ± 13.2	17.8 ± 13.3	0.163
Degenerative index	14.7 ± 8.0	13.3 ± 6.4	0.033*
Prevalence of NP on the examination day (%)	5 (1.8)	70 (14.7)	0.479
Prevalence of NP in the preceding 12 months (%)	12 (4.2)	98 (20.5)	0.181
Prevalence of SS on the examination day (%)	8 (2.8)	178 (37.3)	0.008^#^
Prevalence of SS in the preceding 12 months (%)	20 (6.4)	292 (61.2)	0.315
VAS for NP on the examination day (mm)	29.4 ± 15.4	35.7 ± 22.4	0.734
VAS for NP in the preceding 12 months (mm)	24.0 ± 14.6	42.4 ± 24.1	0.009*
VAS for SS on the examination day (mm)	39.8 ± 25.9	40.0 ± 21.3	0.987
VAS for SS in the preceding 12 months (mm)	35.9 ± 22.0	39.8 ± 22.0	0.382

The values of age, BMI, sagittal alignment at C2–C7, degenerative index, and VAS for neck symptoms are the mean ± SD

*NP* neck pain, *SS* stiff shoulder

*P* values below 0.05* indicate a significant difference between males and females, using the Mann–Whitney *U* test. *P* values below 0.05^#^ indicate a significant association between the prevalence rate of neck symptoms, using the χ^2^ test

The prevalence rate of neck pain on the day of examination and in the preceding 12 months was 1.8 and 4.2 % in 284 males and 14.7 and 20.5 % in 477 females, respectively, with no significant difference between the genders (*P* = 0.479 and *P* = 0.181). The VAS for neck pain on the examination day was 29.4 ± 15.4 in males and 35.7 ± 22.4 in females, which was not a significant difference (*P* = 0.734). The VAS for neck pain in the preceding 12 months was 24.0 ± 14.6 in males and 42.4 ± 24.1 in females; the score for females was significantly higher than that for males (*P* = 0.009). The prevalence rate of stiff shoulder on the examination day was 2.8 % in 284 males and 37.3 % in 477 females; the rate for females was significantly higher (*P* = 0.008). The prevalence of stiff shoulder in the preceding 12 months was 6.4 % in 284 males and 61.2 % in 477 females, which was not a significant difference (*P* = 0.315). The VAS for stiff shoulder on the examination day was 39.8 ± 25.9 in males and 40.0 ± 21.3 in females, which was not significantly different (*P* = 0.987). The VAS for stiff shoulder in the preceding 12 months was 35.9 ± 22.0 in males and 39.8 ± 22.0 in females, which was not significantly different (*P* = 0.382).

### Relationship between roentgenographic findings and age or the VAS for neck symptoms (Tables 3, 4)

Significant correlation between the sagittal alignment of C2–C7 and the degenerative index in males and females was seen (*r* = −0.242, *P* < 0.001 or *r* = −0.222, *P* < 0.001) (Table 3). There was also significant correlation between the sagittal alignment of C2–C7 or the degenerative index and age in females (*r* = 0.204, *P* < 0.001 or *r* = 0.355, *P* < 0.001). Significant correlation between the sagittal alignment of C2–C7 and the VAS for neck pain in the preceding 12 months was seen in females (*r* = −0.154, *P* = 0.011) (Table 3). After adjusting for age, a significant correlation between the sagittal alignment of C2–C7 and the degenerative index was seen in females (*r* = −0.356, *P* < 0.001), which was also true between the degenerative index and the VAS for neck pain on the examination day or in the preceding 12 months (*r* = 0.141, *P* < 0.024 or *r* = 0.187, *P* < 0.003) (Table 3). There were no significant differences on the examination day or in the preceding 12 months in the prevalence rate of neck pain or stiff shoulder among the sagittal alignment groups in males or females (Table 4) or in the VAS for neck pain or stiff shoulder among the same groups (Table 4).

**Table 3:** Relationship between the sagittal alignment at C2–C7 or the degenerative index and age or neck symptoms

	Sagittal alignment of C2–C7			Degenerative index		
	*r*	*P* value	*N*	*r*	*P* value	*N*
Male						
Degenerative index	−0.242	<0.001*	282	–	–	–
Age	0.062	0.298	282	0.502	0.000	282
VAS for NP on the examination day	−0.030	0.861	36	0.235	0.167	36
VAS for NP in the preceding 12 months	0.003	0.987	35	0.115	0.510	35
VAS for stiff shoulder on the examination day	−0.014	0.936	36	0.221	0.194	36
VAS for stiff shoulder in the preceding 12 months	−0.163	0.343	36	−0.045	0.797	36
Female						
Degenerative index	−0.222	<0.001*	479	–	–	–
Age	0.204	<0.001*	479	0.355	<0.001*	479
VAS for NP on the examination day	−0.032	0.518	410	0.046	0.351	410
VAS for NP in the preceding 12 months	−0.154	0.011*	270	0.101	0.098	270
VAS for SS on the examination day	−0.055	0.266	410	0.022	0.659	410
VAS for SS in the preceding 12 months	−0.066	0.217	352	0.028	0.597	352
Partial correlation coefficient (age adjusted)	*r*	*P* value	*df*	*r*	*P* value	*df*
Male						
Degenerative index	−0.270	0.122	32	–	–	–
VAS for NP on the examination day	−0.247	0.159	32	0.213	0.225	32
VAS for NP in the preceding 12 months	−0.110	0.537	32	0.149	0.399	32
VAS for SS on the examination day	−0.109	0.539	32	0.243	0.167	32
VAS for SS in the preceding 12 months	−0.278	0.111	32	0.248	0.158	32
Female						
Degenerative index	−0.356	<0.001*	255	–	–	–
VAS for NP on the examination day	−0.050	0.428	255	0.141	0.024*	255
VAS for NP in the preceding 12 months	−0.073	0.242	255	0.187	0.003*	255
VAS for SS on the examination day	−0.067	0.282	255	0.116	0.064	255
VAS for SS in the preceding 12 months	−0.044	0.486	255	0.104	0.095	255

The relationship between the sagittal alignment at C2–C7 or the degenerative score and age, VAS for neck symptoms were analyzed by Spearman’s rank correlation (unadjusted) and partial correlation analysis (age adjusted)

*r* correlation coefficient, *df* degrees of freedom

*P* values below 0.05* indicate significance

**Table 4:** Association between the sagittal alignment of C2–C7 and neck symptoms

	Straight			Lordotic			Kyphotic			ANCOVA
	Mean (age adjusted)	SEM	*N*	Mean (age adjusted)	SEM	*N*	Mean (age adjusted)	SEM	*N*	*P* value
Male										
Prevalence of NP on the examination day (%)	−0.06	0.37	1	0.14	0.06	35	–	–	0	0.583
Prevalence of NP in the preceding 12 months (%)	−0.01	0.50	1	0.34	0.08	35	–	–	0	0.491
Prevalence of SS on the examination day (%)	0.03	0.44	1	0.23	0.07	35	–	–	0	0.669
Prevalence of SS in the preceding 12 months (%)	1.24	0.49	1	0.54	0.08	35	–	–	0	0.167
VAS for NP on the examination day (mm)	−2.10	12.05	1	4.26	1.99	35	–	–	0	0.607
VAS for NP in the preceding 12 months (mm)	–	–	0	8.23	2.44	35	–	–	0	–
VAS for SS on the examination day (mm)	3.17	21.28	1	9.00	3.51	35	–	–	0	0.789
VAS for SS in the preceding 12 months (mm)	44.49	23.29	1	19.21	3.85	35	–	–	0	0.293
Female										
Prevalence of NP on the examination day (%)	0.20	0.05	45	0.16	0.02	355	0.00	0.10	15	0.197
Prevalence of NP in the preceding 12 months (%)	0.24	0.06	45	0.25	0.02	355	0.21	0.11	15	0.921
Prevalence of SS on the examination day (%)	0.43	0.07	45	0.43	0.03	355	0.35	0.13	15	0.817
Prevalence of SS in the preceding 12 months (%)	0.54	0.07	45	0.60	0.03	353	0.42	0.12	15	0.295
VAS for NP on the examination day (mm)	9.24	2.41	45	5.72	0.86	351	0.13	4.33	14	0.156
VAS for NP in the preceding 12 months (mm)	24.74	5.19	22	13.98	1.57	238	4.51	7.68	10	0.060
VAS for SS on the examination day (mm)	15.18	3.53	45	16.27	1.26	351	11.77	6.33	14	0.762
VAS for SS in the preceding 12 months (mm)	31.51	4.38	34	30.67	1.46	307	27.39	7.70	11	0.897

	Straight vs lordotic	Straight vs kyphotic	Lordotic vs kyphotic							
	*P* value	*P* value	*P* value							
Male										
Prevalence of NP on the examination day (%)	–	–	–							
Prevalence of NP in the preceding 12 months (%)	–	–	–							
Prevalence of SS on the examination day (%)	–	–	–							
Prevalence of SS in the preceding 12 months (%)	–	–	–							
VAS for NP on the examination day (mm)	–	–	–							
VAS for NP in the preceding 12 months (mm)	–	–	–							
VAS for SS on the examination day (mm)	–	–	–							
VAS for SS in the preceding 12 months (mm)	–	–	–							
Female										
Prevalence of NP on the examination day (%)	1.000	0.224	0.293							
Prevalence of NP in the preceding 12 months (%)	1.000	1.000	1.000							
Prevalence of SS on the examination day (%)	1.000	1.000	1.000							
Prevalence of SS in the preceding 12 months (%)	1.000	1.000	0.490							
VAS for NP on the examination day (mm)	0.510	0.200	0.617							
VAS for NP in the preceding 12 months (mm)	0.144	0.090	0.685							
VAS for SS on the examination day (mm)	1.000	1.000	1.000							
VAS for SS in the preceding 12 months (mm)	1.000	1.000	1.000							

The VAS values for neck symptoms are the mean (age adjusted) ± SEM

The association between the sagittal alignment of C2–C7 and neck symptoms were analyzed by ANCOVA (age adjusted). A multiple comparison with the Bonferroni method was conducted after adjusting for age using analysis of covariance in females. *P* values below 0.05* indicate significance

## Discussion

To our knowledge, this is the first community-based survey for associations between roentgenographic findings of the cervical spine and neck symptoms, adjusting for age. The present study revealed prevalence rates of neck pain on the day of examination and in the preceding 12 months of 1.8 and 4.2 % in 284 males and 14.7 and 20.5 % in 477 females. In Norway, Bovim et al. [2] observed in a random sample of 10,000 persons aged 18–67, a prevalence of 13.8 %. In a similar study in Finland, Makela et al. [3] discovered neck pain in 9.5 % of male and 13.5 % of female participants. Thus, the prevalence rate of neck pain in our study was similar to that in previous reports. Okada et al. [17] investigated asymptomatic healthy volunteers, who were followed for 10 years to longitudinally evaluate the relationships between the development of stiff shoulder and sagittal alignments of the cervical spine. Stiff shoulder was found in 29.6 % of the subjects over the 10 years. Stiff shoulder was significantly more frequent in women (45.2 %) than men [17] In our study, the prevalence rate of stiff shoulder on the examination day and in the preceding 12 months was 2.8 and 6.4 % in 284 males and 37.3 and 61.2 % in 477 females; the rate for females on the day of examination was significantly higher than that for males. Although more focused investigation is needed, these results may have implications for current epidemiological issues. Furthermore, the VAS for neck pain in females was significantly higher than for males in our study. The gender difference might be explained if the males were simply more stoic and therefore reported lower scores [9].

In our study, the degenerative index correlated with the VAS for neck pain in females after adjusting for age (Table 3). Marchiori et al. performed a cross-sectional study of 700 consecutive patients and examined correlations between cervical radiographic findings of spinal degeneration and neck pain or disability. They identified a relationship between the number of levels of cervical spine degeneration and the chronicity of the complaint or (in woman only) the associated disability [9]. Gore et al. performed a 10-year longitudinal follow-up study of 200 asymptomatic individuals, using plain X-ray at follow-up, in which neck pain was recognized in 15 % of the subjects. They found that subjects with degenerative changes of C6/7 were significantly more likely to develop neck pain in the future [8]. However, other reports have shown that there is no significant relationship between degenerative changes and pain [7, 10, 11]. Neck pain is a condition far too multifactorial to be correlated with alignment and degeneration seen on conventional X-ray alone. This issue has also been investigated numerous times before, and conflicting results exist, again suggesting that X-ray alone is inadequate to be correlated with clinical symptoms. Most cross-sectional studies on asymptomatic volunteers have shown that degenerative changes in the intervertebral discs are frequently observed on MRI, suggesting that such changes often are not associated with clinical symptoms [6, 19–21]. However, Okada et al. [22] reported a longitudinal study of the cervical spine in healthy volunteers, in which subjects who developed clinical symptoms during a 10-year follow-up, including neck pain and stiff shoulder, demonstrated significantly faster progression of structural disc degeneration on MRI. Thus, the progression of structural changes over a long period is likely to correlate significantly with clinical symptoms.

With age, the number of subluxations and the incidence and severity of degenerative changes increases [7, 8]. Therefore, we analyzed the association between roentgenographic findings of the cervical spine and neck symptoms after adjusting for age. Significant correlation between the degenerative index and VAS for neck pain was seen in females but not males. This may have been because there were fewer degrees of freedom in the males (*df* = 32) compared with females (*df* = 255). In addition, although the degenerative index was significantly higher in males than females, the VAS for neck pain was significantly higher in females than males.

We found no association between the sagittal alignment of C2–C7 and neck symptoms in males or females after adjusting for age (Table 3). Regarding the relationship between sagittal alignment and neck symptoms, Harrison et al. [14] used elliptical and circular modeling to determine the geometric shape of the path of the posterior bodies of C2–C7 in normal, acute pain, and chronic pain subjects. The mean cervical lordosis for all groups could be closely modeled with a circle, and the pain groups had hypolordosis and larger radiuses of curvature compared with the normal group. On the other hand, Grob et al. [15] investigated the association between cervical spine curvature and neck pain in 107 volunteers older than 45. They reported that in the group with neck pain, there was no association between any of the clinical characteristics (duration, frequency, intensity of pain, radiating pain, sensory or motor disturbances, disability, or medical treatment) and the global cervical curvature or segmental angles. Although these results, which are similar to those of our study, were from a general community population, a longitudinal study of the cervical spine following the same individuals may provide clues to the underlying cause of clinical symptoms.

There were some limitations in our study. First, the chronicity of symptoms was not evaluated. The progression of structural changes over a long time period is likely to correlate significantly with future clinical symptoms. Second, the investigation was performed in a limited geographical region, a farming village district that may not be representative of Japan as a whole. Third, there are other causes of spinal pain, including the role of facet degeneration, lateral recess stenosis, and foraminal stenosis in cervical spinal pain. Nevertheless, the current results should be considered in the differential diagnosis and prevention of non-specific neck symptoms.

## Conclusion

Although the sagittal alignment of the cervical spine was not associated with neck symptoms, degenerative change was associated with the severity of neck pain in females.
